# Patient self-assessment and virtual visit-based treatment decisions in rheumatoid arthritis: results from the multicentre Telemedicine in Rheumatoid Arthritis trial

**DOI:** 10.1016/j.ero.2025.06.004

**Published:** 2025-07-10

**Authors:** Johannes Knitza, Johanna Mucke, Felix Muehlensiepen, Tingting Xiong, Manuel Grahammer, Julia Greenfield, Sebastian Kuhn, Nicolas Vuillerme, Gerhard Krönke, Georg Schett, Ann-Christin Pecher, Martin Krusche

**Affiliations:** 1 Institute for Digital Medicine, School of Medicine, Philipps-Universität Marburg, Marburg, Germany; 2 Department of Internal Medicine, Rheumatology and Immunology, Friedrich-Alexander-Universität Erlangen-Nürnberg and Universitätsklinikum Erlangen, Erlangen, Germany; 3 AGEIS, Université Grenoble Alpes, Grenoble, France; 4 Rheumazentrum Ruhrgebiet, Ruhr-Universität Bochum, Herne, Germany; 5 Department of Rheumatology, University Hospital Düsseldorf, Medical Faculty of Heinrich Heine University, Düsseldorf, Germany; 6 Hiller Research Center, University Hospital Düsseldorf, Medical Faculty of Heinrich Heine University, Düsseldorf, Germany; 7 Center for Health Services Research, Brandenburg Medical School Theodor Fontane, Rüdersdorf, Germany; 8 Division of Rheumatology and Systemic Inflammatory Diseases, III. Department of Medicine, University Medical Center Hamburg-Eppendorf, Hamburg, Germany; 9 Department of Rheumatology and Clinical Immunology, Charité - Universitätsmedizin Berlin, Berlin, Germany; 10 Department of Internal Medicine II, Hematology, Oncology, Clinical Immunology, and Rheumatology, University Hospital Tübingen, Tübingen, Germany

## Abstract

**Objectives:**

The growing shortage of rheumatologists threatens rheumatology care. Patient self-assessment and asynchronous virtual visits may improve efficiency. This study assessed the concordance of face-to-face (F2F) and virtual visit-based treatment decisions in individuals with rheumatoid arthritis (RA), incorporating patient self-assessment.

**Methods:**

This prospective 3-month multicentre trial recruited RA patients across 4 university centres in Germany. Patients used a medical app to document the results of a self-performed C-reactive protein (CRP) test, joint count, therapeutic suggestions, and electronic patient-reported outcomes (ePROs) before their regular F2F consultations and ePROs on a weekly basis in-between consultations. Independent tele-rheumatologists provided treatment recommendations based on these data. The primary outcome was the level of agreement between tele-rheumatologists and patients on treatment decisions (escalation, unchanged, de-escalation), compared with F2F rheumatologists’ decisions. Secondary outcomes included change in patient activation (Patient Activation Measure [PAM]) and in patient-perceived ability to assess their disease activity.

**Results:**

A total of 208 consultations and 114 patients (80.7% female, mean age 51.6 years) were included. Most often (63%), treatment remained unchanged, whereas 24% required escalation and 14% de-escalation. F2F treatment decisions aligned with tele-rheumatologist decisions in 77% of cases and patient suggestions in 67% of cases. The accuracy of tele-rheumatologist decisions was 77% for maintaining treatment, 87% for de-escalation, and 91% for escalation. No significant changes in patient activation and patients’ ability to assess their disease activity were observed.

**Conclusions:**

The study demonstrated substantial agreement between virtual asynchronous and F2F treatment decisions. However, further refinement of asynchronous care models and validation studies is crucial.

**WHAT IS ALREADY KNOWN ON THIS TOPIC**

• The growing rheumatologist shortage jeopardises care, and telemedicine is increasingly employed to mitigate its impact.

**WHAT THIS STUDY ADDS**

• This study demonstrated substantial agreement between virtual asynchronous and face-to-face (F2F) treatment decisions in patients with varying levels of disease activity.
• Most patients were unwilling to fully replace F2F visits with virtual care, though a substantial proportion was willing across all disease activity levels.

**HOW THIS STUDY MIGHT AFFECT RESEARCH, PRACTICE OR POLICY**

• The results support the integration of asynchronous virtual visits into routine rheumatology care by demonstrating substantial agreement with F2F treatment decisions.
• Further research is needed to validate these findings, and reimbursement structures should be adapted to provide adequate incentives for care providers.

Alt-text: Unlabelled box

## INTRODUCTION

Rheumatoid arthritis (RA) is one of the most common inflammatory rheumatic diseases, affecting millions of individuals globally [1]. Although prevalence rates vary geographically, they are generally on the rise, with approximately 1% of the adult population in countries like Germany affected [2]. Early and timely initiation of treatment during the ‘window of opportunity’ is crucial to prevent irreversible joint damage and maximise treatment efficacy [3]. Continuous follow-up assessments are essential to monitor disease activity and adjust treatments in alignment with the treat-to-target strategy [4]. However, the growing shortage of rheumatologists [5] and other healthcare professionals poses significant challenges to delivering high-quality care in rheumatology. As a result, new patient consultations are often delayed by months [6,7], and ongoing therapy assessments are not conducted frequently enough to ensure optimal disease control [8].

The COVID-19 pandemic has necessitated a re-evaluation of traditional healthcare delivery models, accelerating the adoption of telemedicine [9] and leading to the development of the first dedicated ‘points to consider’ for remote care by the European Alliance of Associations for Rheumatology (EULAR) [10]. Telemedicine has proven to be a valuable solution, reducing the burden on both patients and healthcare providers while simultaneously addressing increasing healthcare demands [11].

Although synchronous telemedicine, primarily via video consultations, has reduced the need for patient travel, it has yet to yield significant efficiency gains for healthcare professionals. In contrast, asynchronous virtual visits offer a promising approach to improving efficiency for both patients and providers by enabling more flexible, time-efficient management of care while reducing costs [12]. To enable effective asynchronous remote care, a comprehensive information base is essential. Ideally, these data are generated by patients themselves via self-assessment, to foster patient activation and further reduce the time needed by healthcare professionals [13]. Although prior studies have demonstrated that asynchronous virtual visits can be used to effectively triage new patients [14] and assess the necessity of in-person follow-up visits [15,16], no study has yet comprehensively evaluated the concordance between treatment decisions made through virtual visits and those made during face-to-face (F2F) consultations in rheumatology [17].

This prospective multicentre trial aimed to bridge this gap by evaluating the concordance between F2F treatment decisions and those made by tele-rheumatologists based on patient-generated data in individuals with rheumatoid arthritis.

## METHODS

### Study design and setting

The Telemedicine in Rheumatoid Arthritis (TELERA) study was a prospective, 3-month multicentre trial. The detailed study methodology has been published previously [18]. The study design and protocol were developed in close collaboration with 3 official patient research partners from the German League Against Rheumatism (Deutsche Rheuma-Liga Bundesverband e.V.). These partners provided feedback, contributed edits to the draft version and approved the final study protocol and this manuscript. Ethics committee approval was obtained from all 4 participating centres [# 4442020], which included the rheumatology outpatient units of the University Hospitals in Düsseldorf, Erlangen, Hamburg, and Tübingen (Germany). All procedures were conducted in accordance with relevant guidelines and regulations, including the Declaration of Helsinki.

**Figure 1:**
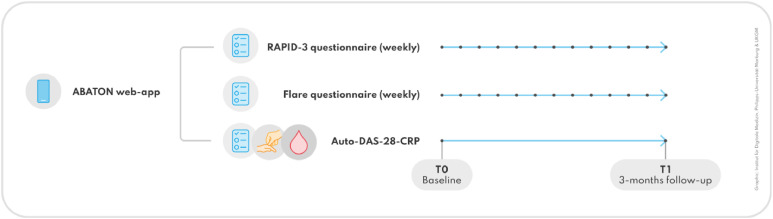
Overview of patient self-assessments collected via the medical app. The Auto-DAS-28-CRP is derived from patient self-assessments, incorporating a semi-quantitative CRP test, joint self-examination, and patient-reported global health status. CRP, C-reactive protein.

Patients used the ABATON medical web app (ABATON GmbH) to document their self-generated data (see Fig 1). This included the Auto-Disease Activity Score (DAS)-28-CRP, which was calculated based on a video-guided joint self-examination (bit.ly/3rlYZTQ), a semi-quantitative capillary-based CRP test (CRP-CHECK-1, VEDALAB), and a patient global health questionnaire. These assessments were completed on the same day just before the regular F2F appointments (T0 and T1). Along with these data, patients also recorded their treatment preference: escalation of therapy, no change in therapy, or de-escalation of therapy. Additionally, on a weekly basis, patients completed a Routine Assessment of Patient Index Data 3 (RAPID-3) questionnaire [19] and responded to a flare question: ‘Did you experience a flare of your disease in the past 7 days?’. Study personnel also uploaded the patients’ general medical history to the rheumatologist dashboard in ABATON to complement the patient-generated data. After the final study visit (T1), patients completed an evaluation questionnaire and had the option to participate in an optional qualitative study [20]. Local rheumatologists did not have access to ABATON for their F2F treatment decisions. Based on the patient’s medical history and the patient-generated data, 2 independent, randomised rheumatologists from other university centres provided their therapeutic recommendations for both T0 and T1.

### Patient inclusion criteria

Inclusion criteria for the study were as follows: (1) a confirmed diagnosis of RA according to the 2010 American College of Rheumatology/EULAR classification criteria, (2) age over 18 years, (3) sufficient proficiency in the German language, (4) possession of smartphone and regular usage and (5) provision of written informed consent.

### Primary outcomes

The primary outcome was the agreement between the therapeutic suggestions made by tele-rheumatologists and patients compared to the gold standard, which was the therapeutic decision made by the local rheumatologist.

### Secondary outcomes

Secondary outcomes included change in patient activation (PAM) [21] and in patient-perceived ability to assess the disease activity since the last in-person consultation (0 to 10 numeric rating scale; 0 = unable to assess my disease activity since my last in-person consultation, 10 = able to assess my disease activity with excellent accuracy since my last in-person consultation). PAM is a validated tool designed to assess a patient’s knowledge, confidence and skills in managing their own health and healthcare on a scale from 0 (lowest activation) to 100 (highest activation). Patient acceptance of CRP self-sampling and willingness to replace routine appointments with virtual visits were assessed using the Net Promoter Score (NPS). The NPS is a market research metric measuring how likely customers are to recommend a product or service to others using one question ‘How likely would you be to recommend…’ on a 0 to 10 numeric rating scale (0 = not at all likely, 10 = extremely likely). The NPS is then calculated by subtracting the percentage of detractors (ratings 0 to 6) from the percentage of promoters (ratings 9 to 10) [22]. Medical app acceptance was assessed using the NPS, and app usability was assessed using the system usability scale (SUS). The SUS is a reliable, standardised tool designed to measure how user-friendly and effective users perceive a system or product. It consists of 10 questions, each rated on a 5-point Likert scale, to assess aspects like ease of use, complexity and confidence when interacting with the system. The SUS score ranges from 0 (lowest) to 100 (highest), with scores above 68 indicating above-average usability and scores over 80 reflecting a high level of usability [23].

### Statistical analysis

Due to the exploratory nature of the trial, no formal sample size calculation was conducted. Instead, an estimated sample size of 120 patients was deemed sufficient to evaluate the primary outcome, accounting for a moderate dropout rate of 20%, resulting in a minimum of 96 patients. A significance threshold of *P* < 0.05 was applied. Descriptive statistics for patient comparisons were presented as the median and interquartile range (IQR), defined as the 25th and 75th percentiles for continuous variables and as absolute numbers (n) and percentages for categorical variables. We assessed the agreement between treatment decisions using Cohen’s κ coefficient. Additionally, we computed sensitivity, specificity, positive predictive value (PPV), negative predictive value and accuracy along with its 95% confidence interval (CI) of tele-rheumatologist treatment decisions using F2F treatment decisions as the reference method. To assess changes in patient activation (PAM) and patients’ ability to judge their disease activity over time, Wilcoxon’s signed-rank test was used. All statistical analyses were performed using R Studio software (version 2024.12.1, R Foundation for Statistical Computing).

## RESULTS

A total of 115 consecutive RA patients were recruited between August 12, 2021, and November 27, 2023, of which 114 patients and 208 consultations were included in the present study. One patient consented but did not complete any questionnaires. Of the 114 patients, 92 (80.7%) were female, the mean age was 51.6 years, the mean disease duration was 8.6 years and the mean travel time to the outpatient consultation was 1.5 hours (Table 1).

**Table 1:** Patient demographics

Characteristic		All patients (*n* = 114)
Demographics		
	Age in years, mean (SD)	51.6 (11.6)
	Sex, female, n (%)	92 (80.7)
	RF, positive, n (%)	71 (62.3)
	ACPA, positive, n (%)	60 (52.6)
	Disease duration, years, mean (SD)	8.6 (8.5)
	Disease duration ≤1 year, n (%)	19 (16.7)
	Travel time, hours, mean (SD)	1.5 (0.8)
Treatment, n, (%)		
	Prednisone	31 (27.2)
	NSAIDs	21 (18.4)
	cDMARDs	67 (58.8)
	bDMARDs	50 (43.9)
	tsDMARDs	20 (17.5)
Disease activity		
	Tender joint count 28, mean (SD)	2.7 (4.0)
	Swollen joint count 28, mean (SD)	1.4 (2.7)
	PhGA (0–10), mean (SD)	2.4 (2.1)
	PtGA (0–10), mean (SD)	3.2 (2.5)
	CRP (mg/l), mean (SD)	5.3 (10.1)
	DAS28 (CRP), mean (SD)	2.7 (2.1)
	DAS28 (CRP) <2.6, n (%)	53 (46.5)
	DAS28 (CRP) 2.6–3.2, n (%)	20 (17.5)
	DAS28 (CRP) >3.2, n (%)	35 (30.7)
	DAS28 (CRP) missing, n (%)	6 (5.2)
	CDAI, mean (SD)	9.6 (9.5)

ACPA, anticitrullinated protein antibodies; bDMARD, biologic disease-modifying antirheumatic drug; CDAI, Clinical Disease Activity Index; cDMARD, conventional disease-modifying antirheumatic drug; CRP, C-reactive protein; DAS28, Disease Activity Score 28; PhGA, Physician Global Asssessment; PGA, Patient Global Assessment; RF, rheumatoid factor; tsDMARD, targeted synthetic disease-modifying antirheumatic drug.

### Concordance of treatment decisions

In most F2F consultations, the treatment regimen remained unchanged (63%, 130/208), whereas treatment escalation occurred in 24% (49/208) of cases and de-escalation in 14% (29/208).

**Figure 2:**
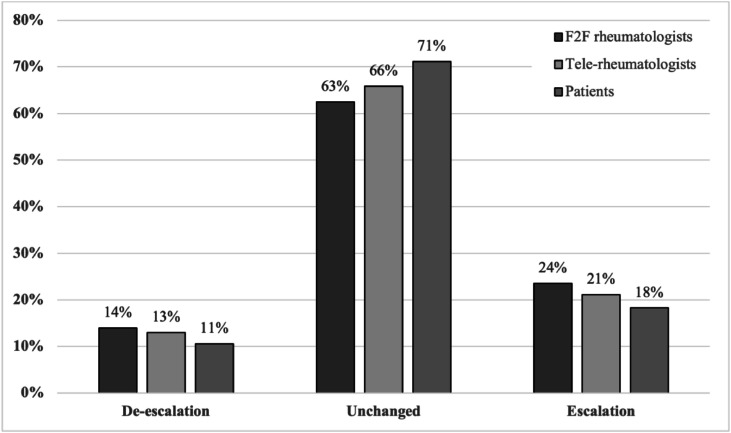
Proportion of treatment decisions made by face-to-face (F2F) rheumatologists, tele-rheumatologists, and patients.

**Figure 3:**
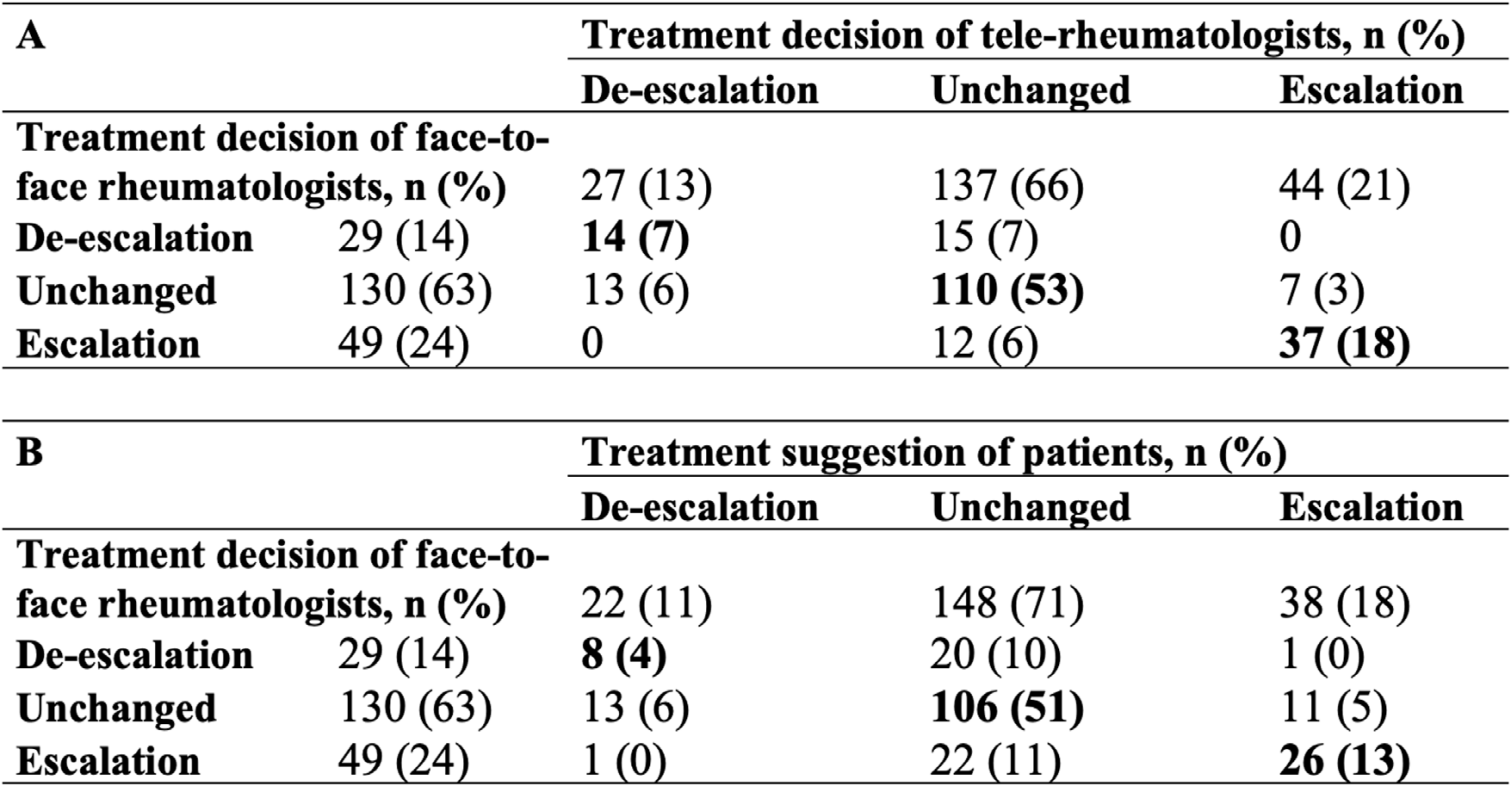
Confusion matrix of (A) tele-rheumatologists’ decisions and (B) patients’ suggestions compared with face-to-face rheumatologists’ treatment decisions. Bold numbers indicate consultations where there was complete agreement on treatment decisions between face-to-face and tele-rheumatologists.

Patients and tele-rheumatologists were less likely to recommend treatment modifications compared with F2F consultations (Fig 2). The agreement between F2F treatment decisions and those made by tele-rheumatologists was substantial, with concordance in 77% (161/208) of cases (κ = 0.64; 95% CI, 0.52–0.75). In contrast, agreement between F2F treatment decisions and patient-recommended changes was fair, with concordance in 67% (140/208) of cases (κ = 0.40; 95% CI, 0.26–0.54) (Fig 3).

Compared to F2F treatment decisions as the gold standard, tele-rheumatologists demonstrated an accuracy of 77.4% for maintaining treatment, 86.5% for de-escalating treatment, and 90.9% for escalating treatment (Table 2). The concordance of F2F and tele-rheumatologists was highest for escalating treatment (κ = 0.74), followed by treatment maintenance (κ = 0.51) and de-escalation of treatment (κ = 0.42).

**Table 2:** Concordance of virtual visits in determining treatment decisions using face-to-face consultation as the reference method

Treatment decision	Sensitivity (95% CI)	Specificity (95% CI)	Accuracy (95% CI)	PPV (95% CI)	NPV (95% CI)	Cohen’s κ
De-escalate	48.3% (29.4%, 67.5%)	92.7% (87.9%, 96.1%)	86.5% (81.1%, 90.9%)	51.9% (31.9%, 71.3%)	91.7% (86.7%, 95.3%)	0.42
Unchanged	84.6% (77.2%, 90.3%)	65.4% (53.8%, 75.8%)	77.4% (71.1%, 82.9%)	80.3% (72.6%, 86.6%)	71.8% (59.9%, 81.9%)	0.51
Escalate	75.5% (61.1%, 86.7%)	95.6% (91.1%, 98.2%)	90.9% (86.1%, 94.4%)	84.1% (69.9%, 93.4%)	92.7% (87.6%, 96.2%)	0.74

NPV, negative predictive value; PPV, positive predictive value.

### Effects of patient self-assessment

No significant changes were observed in patient activation (PAM) from baseline to 12 weeks (median [IQR]: T0 = 67.8/100 [58.1–75.0]; T1 = 65.5/100 [60.6–72.5]; *P* = 0.72). Similarly, there was no significant change in patients’ ability to assess their disease activity since the last visit (median [IQR]: T0 = 6/10 [[3], [4], [5], [6], [7], [8]]; T1 = 7/10 [[4], [5], [6], [7], [8], [9]]; *P* = 0.37).

### Patient acceptance and app usability

Patient willingness to replace routine appointments with virtual visits was low, with an NPS of −17.9% (27.4% promoters; 45.3% detractors). However, patients willing to replace routine visits with virtual visits were present across all disease activity levels (DAS28-CRP). Among patients with low disease activity, the NPS was –18.9% (24.5% promoters; 43.4% detractors); for moderate disease activity, –28.6% (23.8% promoters; 52.4% detractors); and for high disease activity, –22.9% (25.7% promoters; 48.6% detractors). CRP self-sampling received an NPS of +7.3% (43.8% promoters; 36.5% detractors). The monitoring app was well received, with a mean SUS score (SD) of 84.3 (14.9) and an NPS of +14.3% (39.8% promoters; 25.5% detractors).

## DISCUSSION

This prospective multicentre trial demonstrated that treatment decisions for patients with rheumatoid arthritis can be made with reasonable confidence using virtual visits. To the best of our knowledge, this is the first study to assess the concordance of fully asynchronous virtual visit-based treatment decisions in rheumatology. Key strengths of this study include its real-world, multicentre design and the inclusion of a substantial proportion of patients with high disease activity and short disease duration.

A pivotal study by Piga et al [24] reported high reliability of video consultations compared with F2F consultations for treatment decisions across a diverse rheumatic patient population, including RA patients. Their total agreement rate of 84% was slightly higher than the 77% observed in our study. Notably, the findings of the present study align with those of Piga et al, who reported the lowest sensitivity for treatment de-escalation. Importantly, treatment modification decisions (both escalation and de-escalation) made by tele-rheumatologists exhibited a high negative predictive value, indicating that changes were implemented only when truly necessary. The superior results of video consultations are likely due to the additional information gained through direct patient interaction. Although conducted in a different clinical context, a study in paediatric otolaryngology showed that allowing clinicians to ask follow-up questions significantly improved treatment concordance [25]. Building on this, future studies should explore the integration of follow-up question mechanisms within clinical workflows. In particular, an asynchronous chat-based approach could be investigated to determine its effectiveness in optimising treatment concordance while preserving the flexibility advantages of asynchronous communication. To make this asynchronous telemedicine approach even more scalable, virtual visits could also be performed by rheumatology nurses [26] or general physicians. Evidence suggests that virtual visits by rheumatologists are effective in managing gout [27], and we consider it likely that this approach could also be extended to general physicians. Furthermore, assessing the preferences of both patients and healthcare professionals could provide valuable insights into the feasibility and clinical implementation of such an approach.

Piga et al [24] highlighted that disagreements often stemmed from the inability to perform a physical examination remotely. In this regard, our findings demonstrated that patient self-assessment could contribute to overcome this barrier. Emerging technologies, such as smartphone-based joint examinations [28], show promise in further enhancing virtual rheumatology patient management. Notably, the treatment deviations observed in our study may not be solely attributed to the asynchronous virtual format. Rather, they may reflect the inherent variability in rheumatologists’ decision-making, as demonstrated in a prior study [29] using 10 real-world RA vignettes, where no consensus was reached for a single case.

Consistent with qualitative findings [20], quantitative patient feedback indicated a general appreciation for self-assessment and the use of the medical app. However, the majority of patients were hesitant to fully replace in-person consultations with virtual visits. Importantly, patients willing to replace routine visits with virtual visits were present across all disease activity levels. A key reason for this reluctance may be that study participation did not alter patient care; patients received no feedback on their self-assessment results, nor did these results influence treatment decisions. In contrast, a similar study in axial spondyloarthritis, where self-sampling and ePRO results were discussed with patients via phone consultation to determine the need for in-person visits, achieved significantly higher patient acceptance [16]. Likewise, patient satisfaction in the video consultation study by Piga et al was notably high. These findings underscore the critical role of effective patient communication in telemedicine and highlight the need to integrate meaningful patient–clinician interactions into virtual care models to enhance acceptance and engagement.

This trial has several limitations. First, this study deliberately lacked direct patient–clinician interaction, which may have influenced treatment concordance and patient acceptance. Future research could enable tele-rheumatologists to gather additional clinical information if needed. Second, although the study included multiple university centres, the findings were from a single country and may not be fully generalizable to broader rheumatology settings, such as private practices or community clinics. Third, despite recruiting a diverse RA population, selection bias cannot be ruled out, as participation requires digital literacy and access to a smartphone. Additionally, the number of screened patients was not recorded, limiting a statement regarding suitability of this approach, and no formal a priori sample size calculation was conducted. This may limit the precision and generalisability of the agreement estimates, as the study was not powered to detect a specific level of agreement. Furthermore, the adoption of virtual visits remains limited in many countries due to the lack of reimbursement policies. Cost-effectiveness studies such as TeleSpA [15] are needed to address these gaps. Finally, this study classified treatment decisions into 3 categories to facilitate comparison with previous research [24]. However, real-world treatment is more complex, involving variations in dosages and medication choices. Nevertheless, this approach could function as a triage system to identify patients in need of therapeutic adjustments, prioritise F2F visits for those with active disease and support virtual consultations for treatment de-escalation. Further research is required to assess the effectiveness of this model in clinical practice. Importantly, the primary objective should be to expand rheumatology care capacities, with telemedicine serving as a complementary strategy to help mitigate the growing shortage of specialists.

## CONCLUSION

This study underscores the potential of asynchronous virtual visits to enhance efficiency in rheumatology and provide more flexible care options. Although substantial agreement was observed between virtual and F2F treatment decisions, the importance of patient–physician communication and the need for telemedicine-compatible physical examinations became evident. Further research is required to refine remote care models and optimise their integration into clinical practice.

## CRediT authorship contribution statement

**Knitza:** Writing – review & editing, Writing – original draft, Visualization, Validation, Supervision, Software, Resources, Project administration, Methodology, Investigation, Funding acquisition, Formal analysis, Data curation, Conceptualization. **Johanna Mucke:** Writing – review & editing, Supervision, Project administration, Methodology, Investigation, Funding acquisition, Conceptualization. **Felix Muehlensiepen:** Writing – review & editing, Supervision, Conceptualization. **Tingting Xiong:** Writing – review & editing, Validation, Project administration, Data curation. **Manuel Grahammer:** Writing – review & editing, Supervision, Software, Resources, Project administration, Data curation, Conceptualization. **Julia Greenfield:** Writing – review & editing, Visualization, Formal analysis, Data curation. **Sebastian Kuhn:** Writing – review & editing, Validation, Supervision, Resources. **Nicolas Vuillerme:** Writing – review & editing, Supervision, Resources. **Gerhard Krönke:** Writing – review & editing, Supervision, Resources, Project administration, Investigation. **Georg Schett:** Writing – review & editing, Supervision, Resources, Project administration, Investigation. **Ann-Christin Pecher:** Writing – review & editing, Visualization, Validation, Supervision, Resources, Project administration, Methodology, Investigation, Funding acquisition, Formal analysis, Data curation, Conceptualization. **Martin Krusche:** Writing – review & editing, Validation, Supervision, Software, Resources, Project administration, Methodology, Investigation, Funding acquisition, Formal analysis, Data curation, Conceptualization.

## Competing interests

SK is the founder and shareholder of MED.digital GmbH. MG is the CEO of ABATON. JK received speaker fees from Sanofi. Johannes Knitza is an associate editor of ERO.
